# New Approaches for the Discovery of Pharmacologically-Active Natural Compounds

**DOI:** 10.3390/biom9030115

**Published:** 2019-03-23

**Authors:** José L. Medina-Franco

**Affiliations:** Department of Pharmacy, National Autonomous University of Mexico, Mexico City 04510, Mexico; medinajl@unam.com.mx; Tel.: +5255-5622-3899

Natural products continue to be a major source of active compounds. Natural products from different sources have provided a large number of molecules approved for clinical use or that have been used as the starting points of optimization programs [1,2]. Similarly, natural products have inspired the synthesis and development of biologically active molecules [3,4]. However, identifying pharmacologically active natural products in an efficient and systematic manner is not an easy task. To this end, a broad range of experimental and computational approaches have emerged and evolved in recent years, boosted by the progress in the technological advances of screening strategies. In many cases, both experimental and theoretical methods are used in synergy [5,6].

This special issue includes nine papers including eight full articles and one review paper from more than 45 scientists from around the world. The papers illustrate the development and/or application of a broad range of computational and experimental techniques applied to natural product research. As described below, several papers also integrate either the creation or the mining of compound databases and web-based resources open to the scientific community interested in research into natural products.

The issue begins with the article by Prieto-Martínez et al. describing the computational and experimental characterization of the flavonoid amentoflavone as a novel inhibitor of bromodomain 4 [7]. The computational studies were performed with docking using four algorithms. This work is an example of a successful synergistic combination of informatic methods with natural products and experimental validation. The work of González et al. discussed the biological activity of a diterpenoid previously extracted from the octocoral *Pseudopterogorgia acerosa* [8]. The marine natural product inhibited the proteasomal chymotrypsin-like activity of murine macrophages in the presence of lipopolysaccharide, but not in its absence. The authors also conducted docking simulations that provided a hypothesis regarding the inhibitory activity of the chymotrypsin-like activity. Loza-Mejía et al. reported on an in silico study of secondary metabolites isolated from plants of the *Calceolaria* genus as bioinsecticides [9]. The compounds were docked with three molecular targets, namely; acetylcholinesterase, prophenoloxidase, and the ecdysone receptor. The findings of the informatics studies were in good agreement with previously published experimental results. In the same work, the authors concluded that verbascoside is a promising candidate for the development of a multitarget insecticide. Soo Moon et al. presented the results of the synthesis of new derivatives of ginsenosides that are distinctive triterpenoidal saponins considered to be responsible for most of the pharmacological activities of *Panax ginseng* (Korean ginseng). One of the newly synthesized compounds, α-glycosylated ginsenoside F1 had increased solubility, lower cytotoxicity toward human dermal fibroblast cells, and higher tyrosinase activity and ultraviolet A (UVA)-induced inhibitory activity against matrix metalloproteinase-1 than the parent ginsenoside F1. The authors concluded that the new compound has potential interest in cosmetic applications [10]. In their research article, Lu et al. reported the identification of squalene in five alpine grasslands soils from the Tibetan Plateau, which is characterized by high altitude, strong solar radiation, drought, low temperatures, and thin air. To this end, the research team used the pyrolysis gas chromatography–mass spectrometry technique and concluded that the harsh environmental conditions of the Tibetan Plateau seemed to stimulate the biosynthesis of squalene. One of the significances of this work is that squalene is a natural product broadly used in the food, cosmetics, and medical industries because of its antioxidant, antistatic, and anti-carcinogenic properties [11]. Pilón-Jiménez et al. described the construction, curation, and informatic analysis of BIOFACQUIM, a compound database of natural products isolated and characterized in Mexico. The compound database is annotated with the name of the compound, source, and link to the original peer-reviewed paper that describes the characterization and potential biological evaluation. The authors mention that the compound database described in their paper is freely accessible online and will be updated [12]. Chen et al. presented the development of a novel machine learning methodology that enabled the identification of natural products in large compound databases. The algorithm can be further employed to measure the product-likeness of small-molecules and visualize atoms that are part of small molecules that are characteristic of natural products or synthetic molecules. The authors of that work have made the best performing models freely accessible [13]. Sánchez-Salgado et al. discussed a systematic literature search of flavonoids as compounds for the treatment of cholestatic liver disease and reported the results of naringenin as a representative flavonoid in an obstructive cholestasis model. The multidisciplinary team found that naringenin had beneficial effects by improving specific metabolic and liver damage biomarkers [14]. The issue ends with the review by Del Prado-Audelo et al., who reviewed the analysis of the chemical composition and the main mechanisms for brain applications of curcumin. The review paper also covered the application of nanoparticles with curcumin and their extensive health benefit applications [15].

In all, the papers in this special issue illustrate examples of the recent progress on the technological advances and applications of different approaches to identify pharmacologically active natural products. Our aim is that the research presented here contributes to advance the field and further encourages multidisciplinary teams and young scientists and students to further advance the discovery of pharmacologically-active natural compounds.

## References

[1] Newman D.J. (2018). From natural products to drugs. Phys. Sci. Rev..

[2] Rodrigues T., Reker D., Schneider P., Schneider G. (2016). Counting on natural products for drug design. Nat. Chem..

[3] Thomford N., Senthebane D., Rowe A., Munro D., Seele P., Maroyi A., Dzobo K. (2018). Natural products for drug discovery in the 21st century: Innovations for novel drug discovery. Int. J. Mol. Sci..

[4] Yao H., Liu J., Xu S., Zhu Z., Xu J. (2017). The structural modification of natural products for novel drug discovery. Expert Opin. Drug Discov..

[5] Chen Y., de Bruyn Kops C., Kirchmair J. (2017). Data resources for the computer-guided discovery of bioactive natural products. J. Chem. Inf. Model..

[6] Saldívar-González F.I., Gómez-García A., Chávez-Ponce de León D.E., Sánchez-Cruz N., Ruiz-Rios J., Pilón-Jiménez B.A., Medina-Franco J.L. (2018). Inhibitors of DNA methyltransferases from natural sources: A computational perspective. Front. Pharmacol..

[7] Prieto-Martínez F.D., Medina-Franco J.L. (2018). Flavonoids as putative epi-modulators: Insight into their binding mode with BRD4 bromodomains using molecular docking and dynamics. Biomolecules.

[8] González Y., Doens D., Cruz H., Santamaria R., Gutiérrez M., Llanes A., Fernández P.L. (2018). A marine diterpenoid modulates the proteasome activity in murine macrophages stimulated with lps. Biomolecules.

[9] Loza-Mejía M.A., Salazar J.R., Sánchez-Tejeda J.F. (2018). In silico studies on compounds derived from calceolaria: Phenylethanoid glycosides as potential multitarget inhibitors for the development of pesticides. Biomolecules.

[10] Moon S.S., Lee H.J., Mathiyalagan R., Kim Y.J., Yang D.U., Lee D.Y., Min J.W., Jimenez Z., Yang D.C. (2018). Synthesis of a novel alpha-glucosyl ginsenoside f1 by cyclodextrin glucanotransferase and its in vitro cosmetic applications. Biomolecules.

[11] Lu X., Ma S., Chen Y., Yangzom D., Jiang H. (2018). Squalene found in alpine grassland soils under a harsh environment in the Tibetan plateau, China. Biomolecules.

[12] Pilón-Jiménez B.A., Saldívar-González F.I., Díaz-Eufracio B.I., Medina-Franco J.L. (2019). BIOFACQUIM: A Mexican compound database of natural products. Biomolecules.

[13] Chen Y., Stork C., Hirte S., Kirchmair J. (2019). NP-scout: Machine learning approach for the quantification and visualization of the natural product-likeness of small molecules. Biomolecules.

[14] Sánchez-Salgado J.C., Estrada-Soto S., García-Jimenez S., Montes S., Gómez-Zamudio J., Villalobos-Molina R. (2019). Analysis of flavonoids bioactivity for cholestatic liver disease: Systematic literature search and experimental approaches. Biomolecules.

[15] Del Prado-Audelo M.L., Caballero-Floran I.H., Meza-Toledo J.A., Mendoza-Munoz N., González-Torres M., Floran B., Cortés H., Leyva-Gómez G. (2019). Formulations of curcumin nanoparticles for brain diseases. Biomolecules.

